# Experience With Inactivated Polio Vaccine Introduction and the “Switch” From Trivalent to Bivalent Oral Polio Vaccine in the World Health Organization’s Western Pacific Region

**DOI:** 10.1093/infdis/jiw574

**Published:** 2017-06-30

**Authors:** Santosh Gurung, Jennifer B Harris, Abu Obeida Eltayeb, Lee M Hampton, Sergey Diorditsa, Tigran Avagyan, W. William Schluter

**Affiliations:** 1 World Health Organization Western Pacific Regional Office, Manila, Philippines;; 2 Global Immunization Division, Centers for Disease Control and Prevention, Atlanta Georgia; and; 3 UNICEF East Asia and Pacific Regional Office, Bangkok, Thailand

**Keywords:** Inactivated poliovirus vaccine, oral poliovirus vaccine, polio eradication.

## Abstract

The World Health Organization (WHO) Western Pacific Region (WPR) has maintained its polio-free status since 2000. The emergence of vaccine-derived polioviruses (VDPVs), however, remains a risk, as oral polio vaccine (OPV) is still used in many of the region’s countries, and pockets of unimmunized or underimmunized children exist in some countries. From 2014 to 2016, the region participated in the globally coordinated efforts to introduce inactivated polio vaccine (IPV) into all countries that did not yet include it in their national immunization schedules, and to “switch” from trivalent OPV (tOPV) to bivalent OPV (bOPV) in all countries still using OPV in 2016.

As of September 2016, 15 of 17 countries and areas that did not use IPV by the end of 2014 had introduced IPV. Introduction in the remaining 2 countries has been delayed because of the global shortage of IPV, making it unavailable to select lower-risk countries until the fourth quarter of 2017. All 16 countries using OPV as of 2016 successfully withdrew tOPV during the globally synchronized switch from April to May 2016, and 15 of 16 countries introduced bOPV at the same time, with the remaining country introducing it within 30 days. While countries were primarily responsible for self-funding these activities, additional support was provided.

The main challenges encountered in the Western Pacific Region with both IPV introduction and the tOPV-bOPV switch were related to overcoming regulatory policies and challenges with vaccine procurement. As a result, substantial lead time was needed to resolve procurement and regulatory issues before the introductions of IPV and bOPV. As the global community prepares for the full removal of all OPV from immunization programs, this need for lead time and consideration of the impact on national policies should be considered.

## BACKGROUND

The World Health Organization (WHO) Western Pacific Region was the second of the 6 WHO regions to be certified as polio-free in 2000. Though it has had several imported cases of wild poliovirus (WPV) as well as multiple emergences of circulating vaccine-derived polioviruses (cVDPVs), poliovirus transmission has been halted within the specified period, sustaining polio-free status [1, 2]. While the development of cVDPVs from oral polio vaccine (OPV) is rare, cVDPVs have constituted a growing proportion of paralytic polio cases, as the number of WPV cases has diminished [3]. The Western Pacific Region’s last imported WPV case was detected in China in 2011, while its last cVDPV was detected in the Lao People’s Democratic Republic in January 2016 [2, 4].

After the World Health Assembly’s 2012 declaration of polio as a global health emergency, the Global Polio Eradication Initiative (GPEI) developed the Polio Eradication and Endgame Strategic Plan 2013–2018, referred to as “the Endgame Plan,” to outline the actions needed to eradicate polio [5]. In line with this plan, the Western Pacific Region, in coordination with the United Nations Children’s Fund’s (UNICEF’s) East Asia and Pacific Region and other partners, participated in the globally coordinated effort to replace trivalent OPV (tOPV) with bivalent OPV (bOPV) during the period of 17 April to 1 May 2016, commonly referred to as “the switch.” This is the first phase of the eventual withdrawal of all OPV [6]. Trivalent OPV contains all 3 poliovirus serotypes (types 1, 2, and 3), while bOPV contains only types 1 and 3. As type 2 wild poliovirus has been eradicated [7], switching to bOPV should reduce the risk of polio disease caused by cVDPVs originating from the type 2 component of OPV. Between January 2005 and May 2016, 721 cases of polio caused by cVDPVs were reported globally, 94% of which were type 2 cVDPVs (cVDPV2s) derived from the type 2 component of OPV [8]. In the Western Pacific Region, 20 cases of polio caused by cVDPVs have been reported since 2000 [9]. The reported cases include 3 cases of polio caused by type 1 cVDPV (cVDPV1) in the Philippines in 2001, 2 cases of polio caused by cVDPV1 in China in 2004, 2 cases of polio caused by type 3 cVDPV (cVDPV3) in Cambodia in 2005–2006, 2 cases of polio caused by cVDPV2 in China in 2012, and 11 cases of polio caused by cVDPV1 in 2015–16 in the Lao People’s Democratic Republic [9]. Withdrawal of tOPV removes a principal source of immunity against infections with type 2 poliovirus. To minimize risks associated with type 2 live-attenuated vaccine poliovirus, the switch was globally synchronized, and efforts were made to ensure that all tOPV was removed from the cold chain after the switch [10, 11].

In preparation for the switch, the Endgame Plan called for all countries that did not already use inactivated polio vaccine (IPV) in their routine immunization programs to introduce at least 1 dose of IPV into their immunization schedules [5]. One dose of IPV reduces risk by protecting individuals against paralytic polio should they be exposed to cVDPV2 or WPV2 after withdrawal of OPV2, and by priming population immunity for monovalent OPV2 (mOPV2) use in the setting of an outbreak of type 2 poliovirus post-OPV2 cessation [12]. As of late 2014, 20 of the Western Pacific Region’s 37 countries and areas had vaccination schedules that included IPV, either alone or in combination with OPV, while the remaining 17 countries and areas used OPV only and thus needed to introduce at least 1 dose of IPV into the national immunization schedule.

## IPV INTRODUCTION

**Figure 1.**
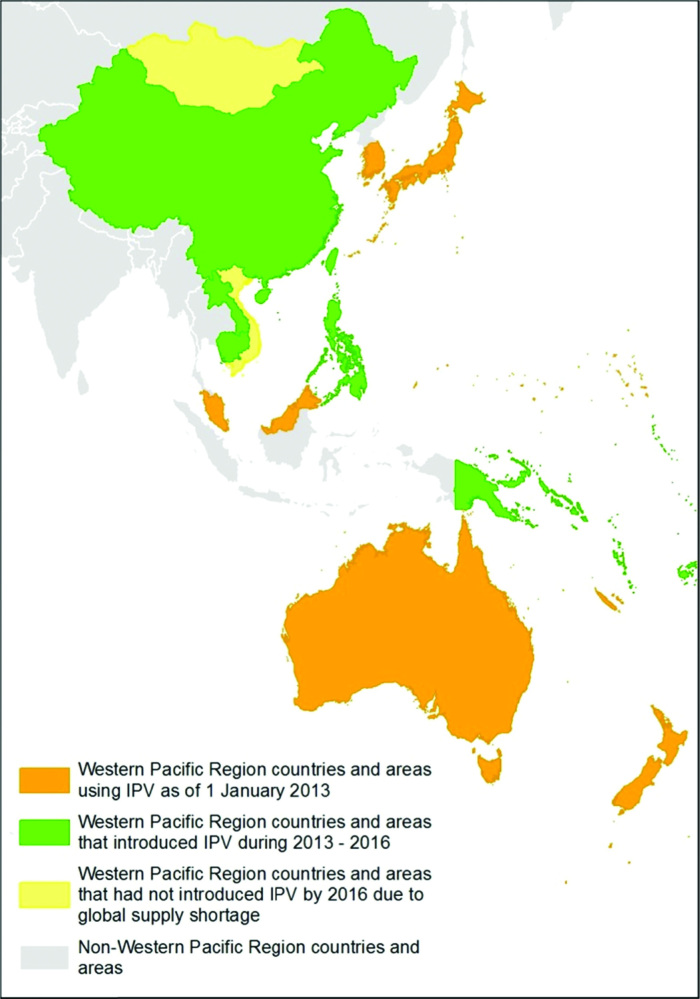
IPV introduction status by country and area, Western Pacific Region—2016 (gray scale).

IPV introduction into the 17 Western Pacific Region countries and areas that did not use IPV as of 2014 began in December 2014 with Beijing Municipality in China (Table 1; Figure 1). The next introductions took place during the second and third quarters of 2015 in a few regions in the Philippines, as well as Papua New Guinea, Kiribati, and Solomon Islands. In the fourth quarter of 2015, IPV was introduced into Cambodia, Cook Islands, Fiji, the Lao People’s Democratic Republic, Nauru, Samoa, Tokelau, Tonga, Tuvalu, and Vanuatu, resulting in 15 countries and areas introducing IPV before the April–May 2016 switch. Because of the global shortfall of IPV that resulted from difficulties in scaling up IPV production faced by the 2 manufacturers supplying GPEI-supported countries, introduction of IPV in 20 countries, including Mongolia and Vietnam, will be delayed until at least the fourth quarter of 2017 [13–15]. While ideally all countries would have introduced IPV before the switch, Mongolia and Vietnam are considered to be low risk for a type 2 cVDPV outbreak after the switch and are classified as Tier 4 (the lowest risk tier of 4 tiers) in GPEI’s risk classification system [16]. (Tier 1 countries are WPV-endemic countries or countries that have reported a cVDPV2 since 2000; Tier 2 countries have reported a cVDPV1/cVDPV3 since 2000 or large/medium countries with DTP3 coverage of less than 80% in the past 3 years [per WUENIC]; Tier 3 countries are those which share a border with Tier 1 countries that reported cases of polio caused by WPV since 2003 or countries that have experienced WPV importation since 2011 [including environmental samples]; Tier 4 countries are countries to which none of these criteria apply.) The IPV shortage has also affected the resupply of IPV to countries that have already introduced IPV. Thirty countries globally are expected to experience stock-outs of IPV nationally before they receive their next supply of IPV, including 8 Tier 4 Pacific Island Countries in the Western Pacific Region: Cook Islands, Fiji, Kiribati, Nauru, Samoa, Solomon Islands, Tonga, and Vanuatu [13].

**Table 1.** IPV Introduction

No.	Countries	Birth Cohort (JRF 2015)	IPV Introduction	DPT3 Coverage (%) (WUNEIC 2015)
1	Cambodia	381245	December 2015	89
2	China	16550000	December 2014 (phase-wise)	99
3	Cook Islands	234	November 2015	99
4	Fiji	20236	December 2015	99
5	Kiribati	3340	June 2015	87
6	Lao PDR	194026	October 2015	89
7	Mongolia	81715	Delayed to 4Q 2017	99
8	Nauru	377	October 2015	91
9	Papua NG	263545	August 2015	62
10	Philippines	2747843	July 2015 (phase-wise)	60
11	Samoa	5915	October 2015	66
12	Solomon Is.	18450	October 2015	98
13	Tonga	2724	December 2015	82
14	Tokelau	30	November 2015 (all IPV-schedule)	100^a^
15	Tuvalu	265	November 2015 (all IPV-schedule)	96
16	Vanuatu	8283	November 2015	64
17	Vietnam	1753504	Delayed to 4Q 2017	97

Abbreviations: DPT3, diphtheria-tetanus-pertussis; JRF, Joint Reporting Forms; Q, quarter; UNICEF, United Nations Children’s Fund; WHO, World Health Organization; WUENIC, WHO/UNICEF estimates of national immunization coverage.

^a^Data is from JRF, not available in the WHO/UNICEF WUENIC.

**Figure 2.**
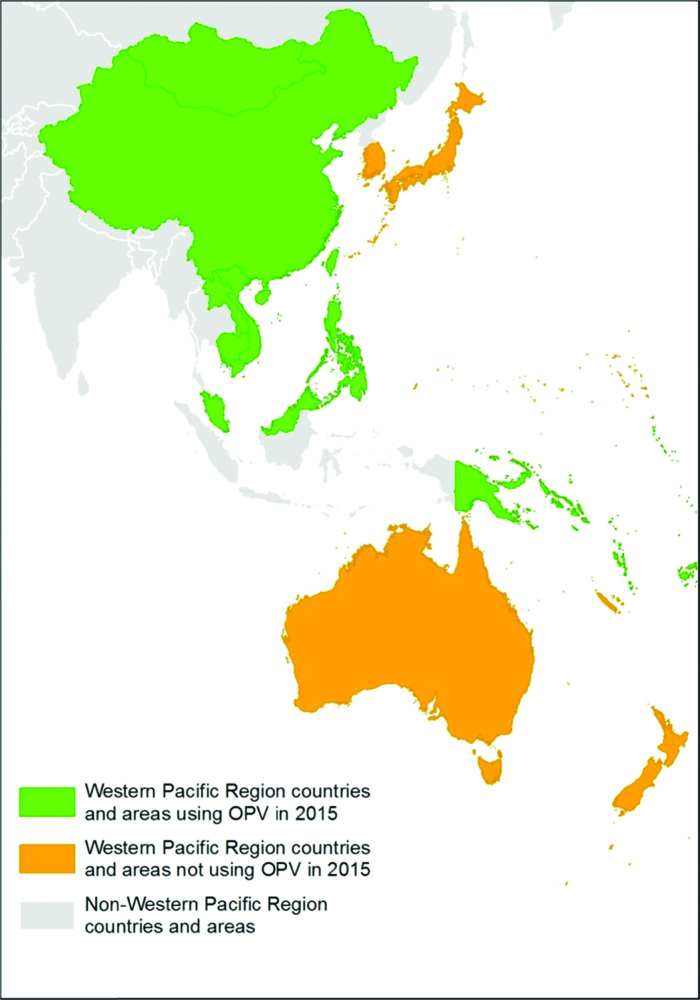
Countries and areas using oral poliovirus vaccine in 2015—Western Pacific Region (gray scale).

IPV introductions were nationwide in 13 of the 15 countries and areas. The 2 remaining countries (China and the Philippines) introduced IPV using phased approaches. At the time of the switch in April–May 2016, China had introduced IPV in 12 of 31 provinces, and the Philippines had introduced it in 7 of 17 regions. The introductions of IPV in some, but not all, provinces of the Philippines and China are of concern, as these countries are considered to be at higher risk for a cVDPV2 outbreak because of previous cVDPV emergence [9, 15, 16]. The Philippines has experienced logistical and administrative delays in IPV introduction but expects to have introduced IPV nationwide by the end of 2016. China planned for nationwide IPV introduction before the switch, but some provinces were delayed because of IPV supply shortages. China produces Sabin IPV for domestic use, but the available quantity has been insufficient to meet national requirements. In response to this shortfall, the Government of China is procuring IPV from Sanofi, as this IPV has already been licensed for use in China. As an additional measure to maximize reach and ensure that children receive at least 1 dose of IPV, the Government of China limited use of standalone IPV to the national immunization program. Standalone IPV could not be sold in the private sector, which generally administers a 4-dose schedule of IPV in a combination vaccine.

To ensure the feasibility of IPV introduction on an accelerated timeline, GPEI provided US$4.36 million to 11 Western Pacific Region countries via grants administered by Gavi, the Vaccine Alliance, and WHO to fund introduction activities, including staff training, cold chain expansion, and social mobilization. The Korea Centers for Disease Control and Prevention provided an additional US$200000 for 4 countries, and WHO provided US$50000. China received support from the Gates Foundation, but this covered only a small portion of their introduction costs related to planning for IPV introduction; the rest, including the cost of the vaccine, was government-financed. UNICEF supported the Pacific islands by procuring IPV supply for 1 year to ensure timely introduction, as government planning and budgeting had already been completed, making it difficult for the countries to allocate funds in a timely way. Concerning IPV procurement in the region, most of the countries procure vaccines through UNICEF’s Supply Division, the Philippines self-procures, and China produces Sabin IPV using domestic manufacturers with self-procurement from international manufacturers as needed.

## tOPV-bOPV SWITCH

As of early 2015, 19 Western Pacific Region countries and areas included 1 or more OPV doses in their routine immunization schedule either alone or in combination with IPV. Malaysia, Tokelau, and Tuvalu moved to all-IPV immunization schedules in November–December 2015, leaving 16 Western Pacific Region countries using OPV in 2016 (Tables 2, 3). These 16 countries engaged in extensive preparations for the switch from tOPV to bOPV planned for April 17–May 1 2016, which allowed all of them to fully discontinue tOPV use during the switch period.

**Table 2.** tOPV-bOPV Switch

No.	Country	Date of Switch	Site Monitoring		Excess of tOPV Disposed (no. of vials)	Method of Disposal
			Total	Monitored		
1	Cambodia	19 April 2016	1369	256	18572	Encapsulation
2	China	1 May 2016	121130	1901	36318169 (doses)	Encapsulation, incineration, Autoclaving
3	Cook Islands	25 April 2016	16	16	0	…
4	Fiji	26 April 2016	184	184	74	Incineration
5	Kiribati	20 April 2016	104	104	0	…
6	Laos	29 April 2016	1231	1098	19470	Open-pit burn and bury, incineration
7	Mongolia	27 April 2016	219	219	8100	Open-pit burn and bury, incineration
8	Nauru	26 April 2016	1^a^	1	0	…
9	Papua New Guinea	18 April 2016	776	185	34989	Encapsulation, incineration
10	Philippines	27 April 2016	2181	301	38188	Encapsulation
11	Samoa	18 April 2016	12	12	4	Incineration
12	Singapore	30 April 2016	…^b^	…^b^	Unknown	…
13	Solomon Islands	25 April 2016	275	104	1152	Incineration, encapsulation
14	Tonga	25 April 2016	38	34	0	…
15	Vanuatu	28 April 2016	80	42	2282	Open-pit burn and bury
16	Vietnam	1 May 2016	11713	11713	37355	Incineration

Abbreviations: bOPV, bivalent OPV; IPV, inactivated polio vaccine; OPV, oral polio vaccine; tOPV, trivalent OPV.

^a^Given the small birth cohort and size of Nauru Island, there is only 1 cold chain store for vaccines.

^b^Singapore provides a sequential IPV-OPV schedule, which includes only 1 dose of OPV administered at age 10–11 years. For switch activities, Singapore focused on 1 distributor and 2 main users of OPV. These 3 sources confirmed cessation of all tOPV distribution and use with replacement by bOPV.

**Table 3.** Switch to All-IPV Schedule

No.	Countries	Date of Switch	Site Monitoring		Excess of tOPV disposed (no. of vials)	Disposal Method
			Total	Monitored		
1	Malaysia	30 December 2015	158	…^a^	3890	Autoclaving
2	Tokelau	1 November 2015	3	3	0	…
3	Tuvalu	2 November 2015	9	9	116	Incineration

Abbreviations: IPV, inactivated polio vaccine; OPV, oral polio vaccine; tOPV, trivalent OPV.

^a^Malaysia’s switch to an all-IPV schedule was conducted in December 2015. As all tOPV stocks were collected and disposed of in December 2015, they did not go through a formal, independent monitoring process following the global switch in April–May 2016.

One of the first events related specifically to preparing for the switch was a “dry run” switch planning workshop, which was organized in Mongolia in July 2015. This workshop was used to test global guidance materials developed for the switch, explore potential barriers to the switch as well as possible solutions, and brief and motivate participating immunization program staff from Mongolia, Lao People’s Democratic Republic, the WHO Regional Office for the Western Pacific, and UNICEF’s East Asia and Pacific Regional Office. After the meeting, staff from the WHO and UNICEF regional offices provided technical support to OPV-using countries to develop their national switch plans. By the end of September 2015, most OPV-using countries had switch plans that included situational analyses, identification of needed preparations, implementation activities, and monitoring and validation components. In most OPV-using countries, the Interagency Coordinating Committee, which coordinates work among government and partners on polio and other vaccine-preventable diseases, served as the national switch management committee, and the National Committee for Certification of Polio Eradication served as the national switch validation committee. The national switch validation committees were responsible for overseeing and evaluating efforts to confirm that all tOPV had been removed from the cold chain [17].

As risk mitigation before the switch, 5 countries (Cambodia, China, Philippines, Vanuatu, and Vietnam) conducted tOPV supplemental immunization activities (SIAs) in areas with suboptimal population immunity or greater risk of importation. Papua New Guinea also administered tOPV for children under age 5 years as a part of its Special Integrated Routine EPI Strengthening Program (SIREP Plus) activities. Lao People’s Democratic Republic conducted 6 rounds of tOPV SIAs between October 2015 and March 2016 in response to the cVDPV1 outbreak there. Four additional SIAs were planned in Lao People’s Democratic Republic from May to December 2016 using bOPV [18].

Countries were asked to conduct tOPV inventories to inform their tOPV procurement and distribution plans before the switch, and most were able to do so. The Philippines encountered the greatest challenge with tOPV supply because it is a self-procuring country and had already ordered enough tOPV to last until November 2016. This agreement could not be cancelled, and thus the April 2016 switch deadline presented special challenges. Furthermore, Philippines’ law prohibits the disposal of potent vaccines. However, the country found a solution through conducting tOPV SIAs in high-risk provinces before the switch, which helped both to increase population immunity to type 2 poliovirus infections and to reduce the country’s stock of tOPV.

All countries using OPV had to procure bOPV and distribute it to the peripheral levels before the switch. All OPV-using countries except China, the Philippines and Vietnam accepted vaccines based on WHO prequalification, and procured bOPV through UNICEF’s Supply Division. Although it is a self-procuring country, GPEI supported the Philippines to secure an initial supply of 4 million doses from a manufacturer producing vaccine already licensed in the Philippines to ensure its timely participation in the switch. Vietnam self-procured, and China began producing bOPV through domestic manufacturers. Vietnam has a policy that requires clinical trials of new vaccines before introduction. During the clinical trial, the Vietnamese authorities conducted a safety study in which 500 children were vaccinated prior to introduction. The first shipment of bOPV did not arrive until April 2016. Nationwide introduction started the first week of June, leaving a 1-month gap between tOPV cessation and bOPV introduction, although the country planned to provide vaccination to children missed during this 1-month interval.

In general, countries were expected to fund switch activities from national budgets. However, funding was made available by GPEI to select low- and lower-middle-income countries that were in GPEI’s Tiers 1 and 2 risk categories. Through this mechanism, US$1.4 million was awarded to 4 Tier 2 Western Pacific Region countries. After these initial funds were disbursed, GPEI opened up applications to other countries that were at risk of not implementing all switch activities without outside support. During this second round of review, 8 additional Western Pacific Region countries were awarded a total of US$296 300 with extensive involvement by the WHO and UNICEF country offices to ensure that program outputs were consistent with GPEI’s Endgame Plan.

All 16 countries using OPV in 2016, including the 4 with delayed or subnational IPV introductions, stopped using tOPV during the globally coordinated period from 17 April to 1 May 2016. All but 1 of these countries introduced bOPV during this period; Vietnam introduced bOPV 1 month later as previously described. All countries except 1 conducted independent monitoring to confirm that tOPV had been withdrawn from the cold chain as recommended by WHO, with participation by 13 international observers deployed to 7 countries that were considered higher risk and/or that requested support with monitoring. By 18 May 2016, all but 2 of the countries in the region had submitted reports from their national switch validation committees confirming that all tOPV had been removed from their vaccine cold chains. While the remaining 2 countries conducted intensified monitoring during the recommended time period immediately after the switch, their monitoring reports went through extensive review and approval processes, thus delaying submission of the reports to the WHO Regional Office for the Western Pacific until July 2016.

## DISCUSSION

The switch from tOPV to bOPV was a success in the Western Pacific Region, with all 16 OPV-using countries withdrawing tOPV during the designated time frame. The switch showed that Western Pacific Region countries could implement activities nationwide with multiple logistic components in a highly coordinated and time-sensitive manner. The WHO Regional Office for the Western Pacific and the UNICEF East Asia and Pacific Regional Office divided country support activities for both IPV introduction and the switch. In some countries, the technical assistance provided included activities to strengthen immunization trainings, outbreak response, and supply and cold chain logistics. However, it was a challenging endeavor, and the long lead time to plan the switch was critical to its success. This lead time allowed countries to fully evaluate the switch, gain necessary internal regulatory and political approvals, plan the highly coordinated logistics, and adapt to challenges prior to the national switch day. This was especially important in China (as it produces its own vaccines) as well as the Philippines and Vietnam, which are large countries that self-procure vaccines. A long lead time for planning and preparation will also be important for the future withdrawal of bOPV.

The effort to introduce IPV in 17 countries between 2014 and 2016 was also a significant challenge. Countries in the Western Pacific Region have vaccine management systems with varying levels of sophistication, and procure vaccines through several mechanisms, including UNICEF’s Supply Division, the Vaccine Independence Initiative [19], and self-procurement directly from vaccine manufacturers, as well as manufacturing vaccines domestically. Because of this, countries encountered diverse challenges in ensuring timely procurement of IPV and bOPV, while managing tOPV consumption in a manner that would minimize both tOPV wastage and the risk for stock-outs before the switch. IPV is an injectable vaccine, and thus countries needed either to add a standalone, injectable vaccine to their national vaccination schedule (thus, increasing the number of injections that infants receive during a vaccination visit) or to incorporate a more expensive combination vaccine [20]. Countries needed time to review options, make costing estimates, and decide how best to incorporate IPV into their vaccination programs.

The changing status of IPV supply posed major problems, forcing several countries to delay IPV introductions by several months, and Mongolia and Vietnam to delay their introductions until after the switch. After rushing to introduce IPV, 8 countries will likely experience stock-outs by late 2016 or early 2017 due to the ongoing global supply constraints. These countries will not be resupplied until the fourth quarter of 2017, which may leave an entire birth cohort of children that will need to be vaccinated once adequate vaccine supplies are made available. As production problems at the 2 manufacturers supplying IPV to GPEI-supported countries was a major cause of these supply problems, this experience underscores the importance of a broad vaccine supply chain with diverse vaccine sources.

The eventual withdrawal of bOPV will remove from routine use an effective tool for preventing poliovirus infection; therefore, it will be essential to ensure that this withdrawal is well executed in a synchronized manner to minimize subsequent risks from VDPVs [10, 11]. A key factor for a successful bOPV withdrawal will be effective long-range planning. Withdrawal dates should be established far ahead of time so that countries, regions, and vaccine manufacturers can plan to use most of their remaining bOPV without experiencing stock-out and can avoid procuring or manufacturing unneeded bOPV. In particular, large countries such as China, the Philippines, and Vietnam that require regulatory approval in addition to or instead of WHO prequalification should be engaged in planning efforts early and frequently, as planning and executing changes to their vaccination programs are more complex and time-consuming than for smaller countries or for countries that import all of their vaccines. In addition to managing the bOPV supply for routine immunization, some countries will need to conduct risk assessments and SIAs prior to withdrawal. Costs of bOPV withdrawal should be estimated at least 1 year beforehand, so that countries can incorporate withdrawal costs in their annual EPI budgets, and partners can plan additional financial support if needed. All countries should establish management structures for bOPV withdrawal (national withdrawal management committees, national validation committees) and develop national withdrawal plans. High-risk or challenging countries should be prioritized to ensure that their plans are developed with adequate time to implement. To encourage the creation of these management structures, regional workshops with the involvement of national EPI managers as well as WHO and UNICEF immunization officers may be effective to disseminate best practices. Ideally, these workshops should be conducted at least 9 months before the withdrawal date.

After the eventual withdrawal of bOPV, strong surveillance and effective outbreak response will remain critical to detect and interrupt any event or outbreak involving WPV or VDPVs [6, 21]. Therefore, effective information, education and communication on strengthening surveillance, outbreak responses, and improving routine immunization coverage should be integrated from the start. Finally, unexpected challenges analogous to the shortage of IPV or the complications some countries faced in securing supplies of bOPV may occur, and flexibility will be needed to meet them. Adjustments to meet these challenges will take time, underscoring the importance of allowing ample lead time for the withdrawal of bOPV.

The switch from tOPV to bOPV and the introduction of IPV in many countries not only brought the Western Pacific Region closer to polio eradication, it also provided a new experience in regional collaboration that demonstrated what the countries of the Western Pacific Region could accomplish when working together. The experience with the switch bodes well not only for the future withdrawal of bOPV but also for future regional efforts against vaccine-preventable diseases.

## Notes

***Acknowledgments.*** The authors would like to thank national immunization program managers, country immunizations staff, and staff from country offices in the WHO Western Pacific Region and UNICEF East Asia and Pacific Region for their valuable contributions in making IPV introduction and the tOPV-bOPV switch successful. This achievement would not have been possible without their tireless dedication. We remain grateful for their willingness to share their individual experiences so that they could be summarized in this manuscript. In addition, we wish to express sincere thanks to Becky Maholland, Division of Emergency Operations Situational Awareness Geographic Information Systems, Centers for Disease Control and Prevention, for the creation of the maps for the article.

***Funding.*** This work was supported by funds from the World Health Organization, UNICEF, and the Centers for Disease Control and Prevention, as well as the Task Force for Global Health on behalf of the Bill and Melinda Gates Foundation.

***Supplement sponsorship.*** This work is part of a supplement coordinated by the Task Force for Global Health with funding provided by The Bill and Melinda Gates Foundation and the Centers for Disease Control and Prevention.

***Potential conflicts of interest.*** All authors: No reported conflicts.
